# Provider attitudes towards a brief behavioral intervention for sexual health in Moldova

**DOI:** 10.1186/s12889-021-11490-5

**Published:** 2021-07-28

**Authors:** Rob Stephenson, Galina Lesco, Viorel Babii, Andrei Luchian, Nataliia Bakunina, Ana Sofia De Vasconcelos, Karel Blondeel, Carlos F. Cáceres, Renée A. Pitter, Nicholas Metheny, Tamar Goldenberg, James Kiarie, Igor Toskin

**Affiliations:** 1 Department of Systems, Populations and Leadership, School of Nursing, University of Michigan, Ann Arbor, USA; 2 Center for Sexuality and Health Disparities, University of Michigan, Ann Arbor, USA; 3 National Resource Centre in Youth Friendly Health Services NEOVITA, Chisinau, Republic of Moldova; 4 UNDP-UNFPA-UNICEF-WHO-World Bank Special Programme of Research, Development and Research Training in Human Reproduction (HRP), Department of Sexual and Reproductive Health and Research, World Health Organization, Geneva, Switzerland; 5 Institute for Leadership and Health Management, I.M. Sechenov First Moscow State Medical University (Sechenov University), Moscow, Russian Federation; 6 Faculty of Medicine and Health Sciences, Ghent University, Ghent, Belgium; 7 Center for Interdisciplinary Studies in Sexuality, AIDS and Society, Universidad Peruana Cayetano Heredia, Lima, Peru; 8 School of Nursing and Health Studies, University of Miami, Coral Gables, FL USA; 9 Carolina Population Center, University of North Carolina at Chapel Hill, Chapel Hill, North Carolina USA

## Abstract

**Background:**

Brief behavioral interventions are seen as an efficient way to improve knowledge, change behavior, and reduce provider stigma regarding sexual health. When grounded in evidence-based behavioral change techniques and delivered using Brief Sexuality-related Communication (BSC) tools, brief behavioral interventions can address client-driven sexual health goals in a single session with their provider. Evidence for the efficacy of brief interventions for creating gains in sexual health comes largely from resource rich settings, and there is a lack of knowledge of how brief interventions can be implemented in the more resource constrained environments of low- and middle-income countries. As a first step in developing a brief intervention to address sexual health issues in Moldova, this paper reports on qualitative data collected from Moldovan providers to understand their attitudes, willingness and perceived barriers to the brief intervention and its implementation.

**Methods:**

Thirty-nine in-depth interviews (IDI) were conducted between February and March 2020, with health providers recruited from three primary health care institutions, two Youth Friendly Health Centers and counselors from three NGOs who work with key populations in Moldova, including health centers selected from two cites - the capital city, Chisinau and from the Comrat Region. The IDI addressed four domains of provider attitudes: 1) attitudes towards the intervention; 2) willingness and motivation to implement the intervention; 3) logistics of providing the intervention and 4) ability to implement the intervention. A coding analysis approach was applied to all interview transcripts.

**Results:**

Providers largely reported being willing to be trained in and implement the brief intervention. Willingness to implement the intervention stemmed from two perceptions: that it would improve the ability of providers to talk with their clients about sex, and that vulnerable groups would benefit from these conversations. However, while there were generally positive attitudes towards the intervention, providers consistently reported structural barriers to their perceived ability to implement the intervention.

**Conclusions:**

While providers reported high levels of initial acceptance of a brief behavioral intervention, care is needed to ensure that brief interventions, and the training of providers on brief interventions, incorporate cultural attitudes and norms around sex, particularly in highly patriarchal settings, and provide opportunities for providers to practice the intervention in ways that address their assumptions and implicit biases.

## Introduction

Brief behavioral interventions aim to identify current or potential health issues or risks and provide a space for critical reflection in which provider and client can work together to motivate clients to change their at-risk behaviors [1]. Brief behavioral interventions delivered in primary care settings can range from 5 to 30 min of counselling, and can be centered on the use of motivational interviewing to stimulate behavior change [2]. Brief behavioral Interventions are seen as efficient ways to improve knowledge, change behavior, [3–9] and reduce provider stigma regarding sexual health [10, 11]. When delivered using Brief Sexuality-Related Communication (BSC) techniques (i.e. the use of goal setting, or critical reflection) [12], brief behavioral interventions can address client-driven sexual health goals in a single session between clients and their healthcare provider. Brief behavioral interventions often use techniques based on motivational interviewing [13]. This client-centered approach enhances intrinsic motivation to change by exploring and by resolving ambivalence and allows healthcare providers to tailor the information available to clients, to improve motivation, and to develop the skills necessary, to critically reflect on and change their risk behaviors [3, 8, 14].

However, much of the evidence on the efficacy and effectiveness of brief behavioral interventions comes from countries with highly developed healthcare systems [8, 15]. Little is known about how brief interventions can be tailored for and implemented in low- and middle-income countries (LMIC), where health care systems may lack the resources for training of providers in motivational interviewing and brief intervention skills. Similarly, there is a dearth of literature on how providers in LMICs view their ability to embed brief interventions into their practice settings and health system environments. Characterized by competing demands for limited resources and a shortage of many types of primary healthcare providers [16–18], the health systems of many LMICs may be initially ill-equipped to widely implement brief interventions, thus requiring important alterations to the content or skills training for brief interventions.

Brief behavioral interventions have demonstrated effectiveness for several sexual health outcomes, including reductions in STIs and/or HIV incidence, across various target groups and in a range of settings, whether they are incorporated into combination prevention programs or delivered as standalone interventions [19–26]. Furthermore, it has been shown that single-session brief behavioral interventions are as effective as more resource-consuming multi-session intensive behavioral interventions for creating gains in sexual health outcomes [27]. However, there is limited knowledge as to how brief interventions that aim to address sexual health issues can be implemented in resource constrained settings.

As a first step to addressing this knowledge gap, the authors developed a brief MI-based behavioral intervention to be delivered by providers in primary health care settings in Moldova, with the aim of improving provider’s skills in motivational interviewing needed for eliciting changes in sexual health behaviors among their clients. As a first step to refining the proposed intervention, we collected qualitative data via in-depth individual interviews (IDI) with providers in Moldova, in order to understand their attitudes and perceived barriers and facilitators to implementing the proposed brief intervention in their current practice. This paper presents the results of a qualitative analysis, reporting on the concerns and beliefs reported by providers in relation to their perceived ability to implement a brief behavioral intervention. Understanding the issues perceived by providers around brief behavioral interventions is instrumental in refining the content, training and delivery mechanisms of these interventions, which are vital for ensuring the interventions can be delivered effectively and with fidelity in resource-constrained primary health care settings.

### The intervention

The brief behavioral intervention is based on techniques of motivational interviewing, developed by Miller and Rollnick [13], defined as a “*directive, client-centered counselling style for eliciting behavior change by helping clients to explore and resolve ambivalence* [13, 28]” and aiming to increase clients’ intrinsic motivation to stimulate change from within rather than being imposed externally [13]. The intervention is designed to be delivered in a single 20–25-min session. During the session, providers will move through a series of eight steps designed to elicit clients’ life goals and current sexual risk behaviors. The eight steps of the intervention are: 1) Understanding the client’s strengths and goals, 2) exploring the client’s recent sexual behavior, risk perception and perceived consequences of risks, 3) using elicit-provide-elicit techniques to provide the client with information on their risks and consequences and asking them to reflect on their risks, 4) exploring benefits of change, 5) buidling a commitment to change and assessing readiness to change, 6) summarizing steps 1–5, 7) focusing on the next steps to achieve sexual health goalsm and 8) working with the client to build an action plan for achieving their sexual health goals. The provider will work with the client to create an individualized action plan comprised of a set of sexual health goals with the aim of improving sexual health, with a specific focus on reducing STIs and unintended pregnancies. An overview of the proposed intervention and proposed protocol for its implementation is published elsewhere [29].

## Study setting

The Republic of Moldova lies in the South-Eastern part of Europe. According to the 2014 census, 75.1% of Moldova’s population reported they were Moldovans, 7.0% Romanians, 6.6% Ukrainians, 4.6% Gagauz, 4.1% Russians, 1.9% Bulgarians, 0.3% Roma, while other ethnicities represented 0.5% of the population. Moldovans, Romanians, Ukrainians, and Gagauz people reside mostly in rural areas, with Russian identifying populations residing mainly in cities. More than half of population (57%) live in rural areas [30]. Since independence from the Former Soviet Republic in 1991, The Republic of Moldova, with a population of approximately 2.6 million in 2020 (compared to more than 4.5 million in 1991), has experienced rapid demographic shifts, driven by massive external migration [30] and reductions in fertility [30]. As a result, since 2017, Moldova has experienced a significant population decrease.

The total fertility rate (TFR) of 2.6 in 1985 declined to 1.77 in 2019 [30]. The TFR is generally higher in rural than urban areas, and peaks in the 20–24-year-old age group. The adolescent pregnancy rate has decreased over the past 10 years, but remains relatively high, with 28.6 births per 1000 women aged 15–19 in 2019 (compared to 41.1 per 1000 in 2014) [31]. Contraceptive use is relatively high, with a contraceptive prevalence rate of 59 and 9.5% of women of reproductive age (WRA) reporting an unmet need for contraception. Use of abortion services remains high in the post-Soviet era, with 30% of women of reproductive age reporting experiencing at least one abortion [32]. Over the past 20 years, the Ministry of Health, in collaboration with the World Health Organization (WHO) have implemented surveillance and prevention services for the monitoring, prevention and treatment of sexually transmitted infections (STIs). As a result, STI rates, including the prevalence of gonorrhea and syphilis have been declining since the early 2000s. In 2000 gonorrhea rates were as high as 50 per 100,000 and syphilis rates at nearly 100 per 100,000, while by 2012, reports show rates had fallen to 31.9 per 100,000 for gonorrhea and 64.6 per 100,000 for syphilis [33]. The prevalence of HIV has remained low, with less than 1% of the population - or 15,000 adults and children - estimated to be living with HIV, but is concentrated among high risk groups of men who have sex with men (MSM) (9%), commercial sex workers (CSW) (4%) and injection drug users (IDU) (13%) [34]. It is estimated that only 64% of individuals living with HIV know their status, 46% are on antiretroviral therapy (ART) and 38% have achieved viral suppression [34]. Levels of engagement in HIV prevention also vary by risk group, with prevalence of condom use estimated at 88% for CSW, 61% for MSM and 26% for IDU [34]. While pre-exposure prophylaxis (PrEP) is available in Moldova, use is reported at less than 1% [35]. These disparities exist in the context of negative social and policy environments for both MSM and CSW [36, 37].

In response to these sexual and reproductive health issues, the Moldovan National Strategic Program in Sexual and Reproductive Health and Rights, 2018–2022, put forth the requirement for sexual and reproductive health services to be available in all primary care settings [38]. To improve access to sexual and reproductive health services among young people, The Republic of Moldova established youth-friendly clinics in all districts between 2002 and 2017 [39]. As of 2019, there are 41 youth friendly Health Centers in Moldova operated by the Ministry of Health. The centers provide young people with counselling, information, sexual and reproductive health services, as well as other medical services.

## Methods

Thirty-nine IDI were conducted between February and March 2020, with health providers recruited from three primary health care institutions, two Youth Friendly Health Centers and counselors from three NGOs who work with key populations. Health centers were selected from two cites: the capital city, Chisinau (i.e. The National Recourse Center in Youth Friendly Health Services NEOVITA, Medico-Territorial Association of the Ryshkan’ sector, and NGOs working with specific populations (i.e. Positivnaya iniciative (for HIV prevention and care), Health and Community Development Centre “AFI”(for female sex workers (FSW)) and GENDERDOC-M (for LGBT individuals) and from Gagauzua Region - District Health center and a Youth Friendly Health Center from Comrat town, Rural Autonomic Health Centre.

After describing an overview of the proposed intervention, the IDI addressed providers’ attitudes towards providing the proposed brief behavioral intervention in their current practice, divided over four domains: 1) overall attitudes towards the intervention; 2) perceived willingness and motivation to implement the intervention; 3) logistics of providing the intervention and 4) perceived ability to implement the intervention. Overall attitudes included exploring issues of perceived utility, perceived appropriateness and safety of the intervention for the clients, and the fit of the intervention within their existing client-provider interactions. The IDI described how the intervention was to be delivered; that it consituted a new approach to how providers would talk to their clients, that it was not a replacement for their current clinical practice, and that the intervention was not intended to be supplemental work. That is, the intervention was described as a framework for how they approached and talked to their clients, and was not an additional part of the routine provider-client visit. Questions regarding intervention logistics included exploring the amount of time that providers felt able to commit to the intervention, the ease of incorporating the intervention into already-existing client-provider interactions, and barriers and facilitators to implementation.

The audio-recorded IDIs lasted between 60 and 90 min, were audio-recorded and were conducted in either Russian or Romanian (the two most widely spoken languages in Moldova), and then translated and transcribed into English. Each transcription was reviewed for accuracy and any identifying information was removed. A coding analysis was applied to all interview transcripts. Codes were discussed and developed by two of the authors (RS and RP). The following interview guide domains were used to develop deductive codes: knowledge of brief interventions, experiences discussing sexual health with clients, comfort discussing sexual health with clients, perceived sexual health needs of clients, and barriers and facilitators to delivering the proposed brief intervention. To identify inductive codes, each theme had sub-themes that emerged from the team’s analyses, discussions, and synthesis of the transcripts. Authors (RS and RP) reviewed each transcript to identify codes and through this process, developed codebooks that included a detailed description of each code, inclusion and exclusion criteria, and examples of the code in use. Given the lack of literature on these topics, codes were inductive and based on emerging themes. Two authors (RS and RP) independently coded each transcript, with discrepancies in coding discussed and resolved at team meetings. The same authors then applied the finalized codes to all transcripts using NVivo software (version 12). The two authors (RS and RP) had several discussions to resolve divergences and reach consensus in the final coding and interpretations.

All procedures performed involving human participants were in accordance with the ethical standards of the institutional and/or national research committee and with the 1964 Helsinki declaration and its later amendments or comparable ethical standards. Ethical approval for this study was obtained from the World Health Organization and Moldovan Ministry of Health. Written informed consent was obtained from all research subjects.

## Results

### Overall attitudes towards the intervention

The majority of the health providers (29 of 39) interviewed had favorable attitudes towards the proposed brief intervention. When the brief intervention was described to them, many health providers noted the potential benefits that the intervention may have for their clients. In particular, those who were already involved in providing mental health services felt that the brief intervention would be most beneficial to “*vulnerable populations*” (i.e. younger clients) and stated the skills used in the brief intervention were very similar to the counseling skills they currently used with their clients “… *almost the same as what we already use in our work with drug users”.* The majority of providers felt that brief intervention described to them in the IDI would fit within the purpose of their existing client-provider interactions, with many noting that it would “*improve*”, “*grow the quality*” and “*make easier and less awkward*” their current interactions. Several participants noted that they already used similar skills to those described in the brief intervention, but each of these also confirmed that they had not received formal training in motivational interviewing, and that “*We are already doing this, but not systematically” and “basically we work like that, we ask questions, and they answer us, but this would help us do it properly”.* There was little direct opposition to the proposed brief intervention, with most providers welcoming the potential for improving their communication skills and recognizing the need for ways to talk about sexual health with their clients.

*“I think it's beneficial, it would be good, at least if they were not explained and informed by the family members at home, by the relatives, I think it would be good when they get to a doctor or a consultation they to be informed, as well as about other social problems that are in our country, and not only in our country, they must be informed”.*

### Perceived willingness and motivation to implement the intervention

While providers were generally in favor of the proposed brief intervention, a small number of providers opposed adoption of the brief intervention (10 of 39). Providers who totally opposed the brief intervention explained their opposition in terms of a lack of time, an already high workload, and a lack of specialized medical knowledge that they felt would limit their ability to provide the brief intervention. Family doctors and nurses more often had reserved attitudes about the possibility of implementing this approach in their practice, perceiving it as an additional overloading task for them– “*Yes, it will be useful, just don’t burden us family doctors with this.”, “I believe that the vector of performance is the family doctor, now the unfortunate family doctor has several tasks, including the field of prevention, different directions and now you are asking him to do even more. And not everyone has the same training and can actually do this”***.** Others expressed doubts, maybe due to a resistance to changing the structure of their consultations from experiences that they have *“First of all, it is not possible to carry out such a consultation during one visit. Secondly, I really don’t even want to” and “I don’t know how I could carry out a short consultation.”*

*“We have to somehow reorganize the work schedule, since no one likes extending the working hours. If the reception hours are increased, the call hours are shortened, thus the working day is lengthened. Nobody wants that”*

### Logistics of providing the intervention

The primary barrier reported by almost all health care providers in the IDI, was the issue of the time needed to deliver the intervention. Providers felt that their time with their clients was already too restricted, and doubted the possibility of adding the brief intervention to their existing appointments, “*It is very welcome, only with the condition of having special time for it. For us, the biggest problem is time”*. Providers were conceptualizing the brief intervention as an addition to existing appointments, requiring more time, and were not viewing the brief intervention as an alternative strategy for delivering care to clients. The issue of time had varied impacts on health care providers’ willingness to adopt the brief intervention. While some felt that with some changes they might be able to incorporate the brief intervention into their appointments, others felt that the time required was totally prohibitive.

*“We agree, but would like to have more time for the consultation.”*

*“I don't think the short consultation will work, because it takes at least 15-20 minutes to approach the given problem. All questions, all steps must be addressed.”*

*“You know, within a short counseling it is not possible to reproduce everything, maybe the client wants something more, but sometimes there is not enough time to do so, but for a client to be satisfied you need to communicate as much as possible with him about the sexually transmitted domain, about birth, depending on the problem the client came with. The more dialog, the better for the client”.*

Participants felt that the brief intervention as described in the IDI would take in the range of 15 to 40 min to deliver (the proposed intervention was described as taking 20 min). Participants felt strongly about keeping the intervention short – around 10–15 min - in order to allow it to be conducted within the parameters of their clinical flow and current appointment times. Several participants, however, were mindful of the sensitivity of the subject matter and suggested the need to find a balance between the intervention content and the amount of time used for the brief intervention.

*“Generally, the topic is very delicate, and, therefore, in order for a person to open up, probably a little more time than for a regular consultation is needed. If usually we have about 15-20 minutes, then probably half an hour on this topic is advisable.”*

Several participants also acknowledged there were administrative barriers to being able to implement the brief intervention. The most commonly cited administrative barriers were “*clinical bureaucracy*”, the need for “*staff training time*”, and concerns over “*client receptiveness*”. In the quote below, one participant brings these issues together, and describes how they believe it would take between “*2 to 5 years*” to implement the brief intervention as they were concerned about needing permission and resources from senior authorities (“*central body*”), and over the need to train staff effectively.

*“If we look at other things already implemented, I think it will take from 2 to 5 years in the best cases. It is very poor communication between the central body, when implementing the regulation, until it reaches the periphery, the order that is issued and disseminated simply by email and the user is not informed about it. Likewise, the staff training process will take extra time, and the public opinion must change, so that the beneficiary also knows about these conditions and sees that they are effective, so that he can come later to make that change that we want to see, then we will see the results at the population level, but the result earlier than 5 years will not be.”*

Similarly, in the quote below, one health care provider believes that although the brief intervention may be implemented as quickly as in 6 months, that competing health demands (i.e. the COVID-19 epidemic in Moldova) and high staff workloads would be a significant barrier to being able to implement the brief intervention.

***“****I think in half a year. Sometimes it can take even longer depending on many circumstances, I said half a year if there are no other situations. Now, if the medical workers are focused on COVID, it is clear that the next 3-4 months they will have a higher workload with all the directions that have remained in the shadows, and the idea of something to be implemented now is excluded. Medical institutions need to start working normally, as it was at least as in January, and then it can be in half a year with some results.”*

### Perceived ability to implement the intervention

When talking about the potential to use motivational interviewing techniques with their clients, providers often contextualized their hesitation in cultural factors around sex and gender. One important cultural factor that was perceived to limit the use of motivational interviewing was a distinction between talking about reproduction and talking about sex. In the quote below, one provider discusses how clients are open to talking about reproduction, but that discussions about sex are often “*taboo*”. In this case, the barrier wasn’t related to the skills involved in the intervention, but more so to the topic of the intervention - the need to talk about sex.

*“Regarding the reproduction, they are more open, since in our country the population wants to have children, they want to have healthy families, to have healthy children and families full of children, 2-3 children, if there is financial possibility, and when it comes to their reproducibility, they speak quite openly. Sometimes they come for a visit as a family, husband and wife come to my office, and there are sometimes questions from our partners, from men that amaze us...”*

Providers noted that sex could be discussed with their clients using the techniques in the described brief intervention, but only for the purpose of discussing reproduction and fertility. Several providers contextualized the reluctance to talk about sex – which they stated was a reluctance among both the provider and client- in “*traditional cultural values*”, which were primarily linked to religion and religiosity.

*“I can’t say that we can only talk on a definite topic ...we are not Muslims, we are mostly Europeans, the religion is the Christianity. But in traditionally Gagauz families, abstinence from sex, that is, they try to preserve some traditions, some concepts of honor, virginity, they are welcome. There are no such strict prohibitions, or to point on somebody with a finger – it is just too hard for people to talk about”*

Strong social norms of patriarchy also emerged in interviews as barriers to implementing the intervention: in the example below, the hesitation in delivering the brief intervention came not in logistical or administrative barriers, but the very topic of talking about sex with male clients was seen as a significant barrier to delivering the intervention.

*“Let's not forget that we are a country with patriarchal traditions, were the main function is often attributed to men, or masculine role it is in direct correlation with men’s sexual health, i.e. if there are any sexual dysfunctions, a man considers himself as a man of a not full value, from a family and social point of view. His role as a man suffers a lot, his self-image suffers in all aspects. So he will not want to talk about any of that”*

In the quote above, one specialist couches their reluctance to talk about sex with their clients in terms of their perceptions that male clients will not talk about sex, due to cultural issues that link sexual health problems among men to diminished masculinity. Similarly, in the quote below, providers note that most providers are not trained to talk about sex with women, and this raised concerns about the ability of providers to provide effective sexual health services to women. Interestingly, as these two quotes illustrate, providers linked men’s inability to talk about sex to the male client, and women’s inability to talk about sex to the skills of the provider.

*“And another issue that needs to be more developed are aspects related to female sexuality, because often we do not have trained specialists in this field, few specialists discuss the given problem, especially discussing this question with the elderly, the questions referring to sexual life and its quality, and with young people about the correlation between contraceptive methods and sexual life, its quality, problems of bullying that arise”.*

In addition to cultural norms of patriarchy and non-discussion of sex, male providers also expressed a lack of comfort with dealing with issues of sexual dysfunction for women and a lack of comfort providing care for female clients more generally.

*“It is easier for me to communicate with guys, I feel easier, I can ask any questions like a guy to a guy. But I also had some problems, but it’s harder to communicate, probably, with the female sex, different people need to be very flexible, for example, when asking questions, because someone can perceive it normally, and someone will be offended or will close. It’s not a problem with men at all, but with women it is”*

While there was a strong pattern of male providers not feeling comfortable talking to female clients about sex, both male and female providers reported concerns over talking to young women about sexual health.

*“Sometimes I feel awkward about the questions the schoolgirls ask, and before answering those question I have to think carefully what to answer and how to answer so that I am correctly understood”*

*“There are girls who even come with their mother and you can't really talk ... because the mother doesn't really like it when you talk to your child about something like that ...”*

*“If there are children under the age of 15, it is still an inconvenience, because, first of all, the consent of the parent is needed, and secondly, he does not understand what we are talking about when it is related to HIV”.*

Providers were generally less comfortable in their ability to provide the brief intervention to CSW or LGBT clients. Providers couched their hesitation in providing the brief intervention to CSW and LGBT clients in terms of a lack of information of the health needs of these populations.

*“I think we need, first of all, to know the specifics of men who have sex with men relations, to have more information for our specifics, that's about it”.*

“*The problem is, we don’t see these people very often – so I am not always sure what they need”*

*“We even had a question – how to ask a woman if she is a commercial sex worker, how can you ask such a question and really how not to humiliate and not to offend her?”*

In the quote above a health care provider reports how they struggle to meet the perceived needs of CSW clients, and that they feel concerned over potentially offending their client by asking them specific questions about their sexual behavior. This lack of confidence in talking with vulnerable populations was a recurring theme among providers, with several noting “*this is not something I see often*” and “*what if they know that I don’t know anything*”. This lack of experience in providing services to vulnerable populations was further articulated as a need to tailor the brief intervention to the specific needs of key populations. In addition to training on the sexual health needs of CSW, adolescent women and LGBT clients, providers often spoke of the need to tailor the brief intervention.

“*It all depends on the target group that uses services of this institution. If this institution works with LGBT people, it is one specificity, if the institution works with drug users – it (the brief intervention) will be different”*

“*Yes, basically, it is about young people. The problem is sexually transmitted infections and unwanted pregnancies that can happen from unprotected and unthought sexual intercourse or poorly thought out in the sense that it results from a risky behavior. It needs to be adapted in expressions, some clients know less, so the information needs to be brought to a slightly lower level, a little more popular, with the use of certain slang expressions that they know, and so on”.*

*“This will have to be very different depending on who we have to deal with – I think the overall structure might be the same, but we will have to be prepared to say very different things to each type of person”*

In addition to discomfort among male providers in talking to women about sex among male providers, and discomfort among all providers in talking about sex with clients from vulnerable groups, providers also noted that they felt they lacked the skills to provide the intervention. Notable exceptions were the small number of providers who were already providing mental health services, who although they had not received training in motivational interviewing, felt that as described, the intervention was similar to how they already communicated with their clients. Among providers who supported the intervention, there was a sense that while they supported it in theory, they were concerned over their ability to implement the brief intervention. Providers mentioned that the motivational interviewing skills were “*very new*” and “*too different*” and were worried over “*feeling embarrassed trying to talk to a client in this way*”. When talking about their perceived skill set, providers most frequently talked about the need for training. Providers wanted opportunities to practice the skills before they delivered them to clients. Providers talked about skills in two main domains: communication skills and medical knowledge on sexual health.

*“If we are shown how to do this, we can do this. But it will need practice – we need teaching – like real training in which we can learn the dos and the don’ts”*

*“It is not like just talking – someone needs to tell us what to say and show us how to say it”*

*“I think also it is important to help us improve our knowledge – so we know the right techniques and procedures that people might need”*

In a similar way that providers felt that the content of the intervention would need to be adapted for vulnerable populations, they also reported that there were training needs specific to delivering the brief intervention to these populations.

*“The training should help us talk to everyone, to gay people and to the young people, each will be very different – and if you don’t know these people, you don’t know how to talk to them”*

## Discussion

Brief behavioral interventions provide an opportunity to train providers on the use of motivational interviewing to address the sexual health needs of their clients. By training providers to shift the ways in which they communicate with their clients – transitioning to working with the client to build a plan – it is possible to achieve sustained behavioral change. In the current study we explored, for the first time, the perceived ability of providers in Moldova to be trained on and implement a brief behavioral intervention in the current practice.

The results of this study are promising: providers largely reported being willing to be trained in and implement the proposed brief intervention, with minimal direct opposition voiced among participants. Willingness to implement the intervention stemmed from two perceptions: that it would improve the ability of providers to talk with their clients about sex, and that clients – particularly vulnerable groups including CSW and MSM – would benefit from these conversations. However, while there were generally positive attitudes towards the intervention, providers consistently reported barriers to their perceived ability to implement the intervention: hence it was often couched as a potentially impactful good idea that couldn’t be implemented realistically. In previous studies, providers have also reported a number of barriers to the delivery of brief interventions, including lack of training or motivation, concerns about their own self-efficacy, and a lack of counselling skills [40, 41]. In the current study, providers’ main concerns centered on structural factors, most notably a lack of belief that the intervention could be delivered within the time limits of a standard session with their clients, and concerns over “*clinical bureaucracy*” that would create a greater need for paperwork to monitor and report on the brief intervention. These concerns are malleable. For example, when the intervention was described to the providers, they often reported that it would take 40 min to implement. This is likely an artifact of it taking longer to describe an intervention than to deliver it. Training of providers should focus on experiential learning, giving providers an opportunity to conduct multiple role plays of the intervention, until they develop the skills and understand how it can be delivered within the proposed 20 min.

While training providers and allowing them opportunities to practice the intervention can likely reduce concerns around the time needed to deliver the intervention, providers also raised a number of structural and institutional barriers to implementation. Providers spoke of institutional bureaucracy as a barrier to being able to successfully implement the brief behavioral intervention. These results demonstrate the training the providers alone may not be sufficient to allow successful implementation. Attention is needed towards structural barriers – for example, working with institutional adminstrators to facilitate understanding of the intervention, and paying equal attention to clients, educating them as to the benefits of participation in the intervention. Changes are necessary across multiple levels - provider, client and institution – to ensure the smooth implementation of the proposed brief intervention.

A substantial body of literature suggests that training health care providers is associated with positive changes in provider intervention behaviors. Providers who have received training are more likely to perform interventions with fidelity [42–44]. Providers interviewed for this study reported the need for extensive training (with the exception of a small number of providers who already provided counseling services), and this result is encouraging as it demonstrates that providers recognize their skill gaps and are willing to be trained to address them. Interestingly, the main barrier to providing the intervention wasn’t the skills required, rather providers were more concerned about the topic – the need to talk about sex. There is a wealth of evidence demonstrating that many providers struggle to talk about sex and sexuality with their clients [45–47]. In a systematic review of qualitative studies of provider-client interactions on sex, Dyer et al. (2013) [48] showed 19 interconnected themes relating to healthcare professionals’ experience of discussing sexuality with clients, including fear about “opening up a can of worms,” lack of time, resources, and training, concern about knowledge and abilities, worry about causing offense, personal discomfort, and a lack of awareness about sexual issues. In this study, providers were particularly concerned about talking about sex with LGBT or CSW clients, and many providers felt that talking with male or young female clients would be difficult. This highlights a significant element of the training for brief interventions – the training must extend beyond teaching providers with skills in motivational interviewing, and must include techniques that address discomfort and implicit biases around talking about sex with clients.

While the results illustrate high levels of willingness and acceptance of the proposed brief intervention, findings should be interpreted within the context of some limitations. Thirty-nine IDI were conducted, and while this is a reasonable sample size for a qualitative study [49], and the authors performed assessments of the extent to which thematic saturation was achieved, the IDI were limited to providers from a small number of clinics in the capital and one region: it may be that providers in smaller clinics or more rural areas have different attitudes to the intervention. Providers had the intervention described to them, but did not see the intervention take place, and it may be that observing an example of the intervention may give providers a more nuanced understanding of the skills involved and may alter their perceptions of the intervention. The research was planned prior to the COVID-19 pandemic but, due to widespread service closures, the IDI had to be conducted online via video-chat. This may have affected participants willingness or openness in discussing some of the attitudes towards the brief intervention.

## Conclusions

Training providers to use motivational interviewing techniques delivered through brief interventions has the potential to shift the ways in which providers talk to their clients about sexual health. While the efficacy of brief interventions is largely established in resource rich settings, work is needed to establish how brief interventions can be tailored for and implemented in resource constrained settings. In this qualitative study with providers in Moldova, we found high levels of initial acceptance of a brief intervention, that were shrouded in structural barriers and concerns around talking about sex with clients. Care is needed to ensure that brief interventions, and the training of providers on brief interventions, incorporate cultural attitudes and norms around sex, particularly in highly patriarchal settings, and provide opportunities for providers to practice the intervention in ways that address their assumptions and implicit biases. The next stage of this research will be to use this qualitative data to refine and pilot test a brief behavioral intervention to be delivered by providers in Moldova, the recognizes the varying skills and comfort levels of providers in talking about sexual health with their clients.

## Data Availability

The datasets analysed during the current study are available from the corresponding author on reasonable request.
